# Supplementary figures and images for: Neutrophil stimulation with citrullinated histone H4 slows down calcium influx and reduces NET formation compared with native histone H4
Source: PLoS One. 2021 May 17;16(5):e0251726. doi: 10.1371/journal.pone.0251726 (PMC8128235; doi:10.1371/journal.pone.0251726)

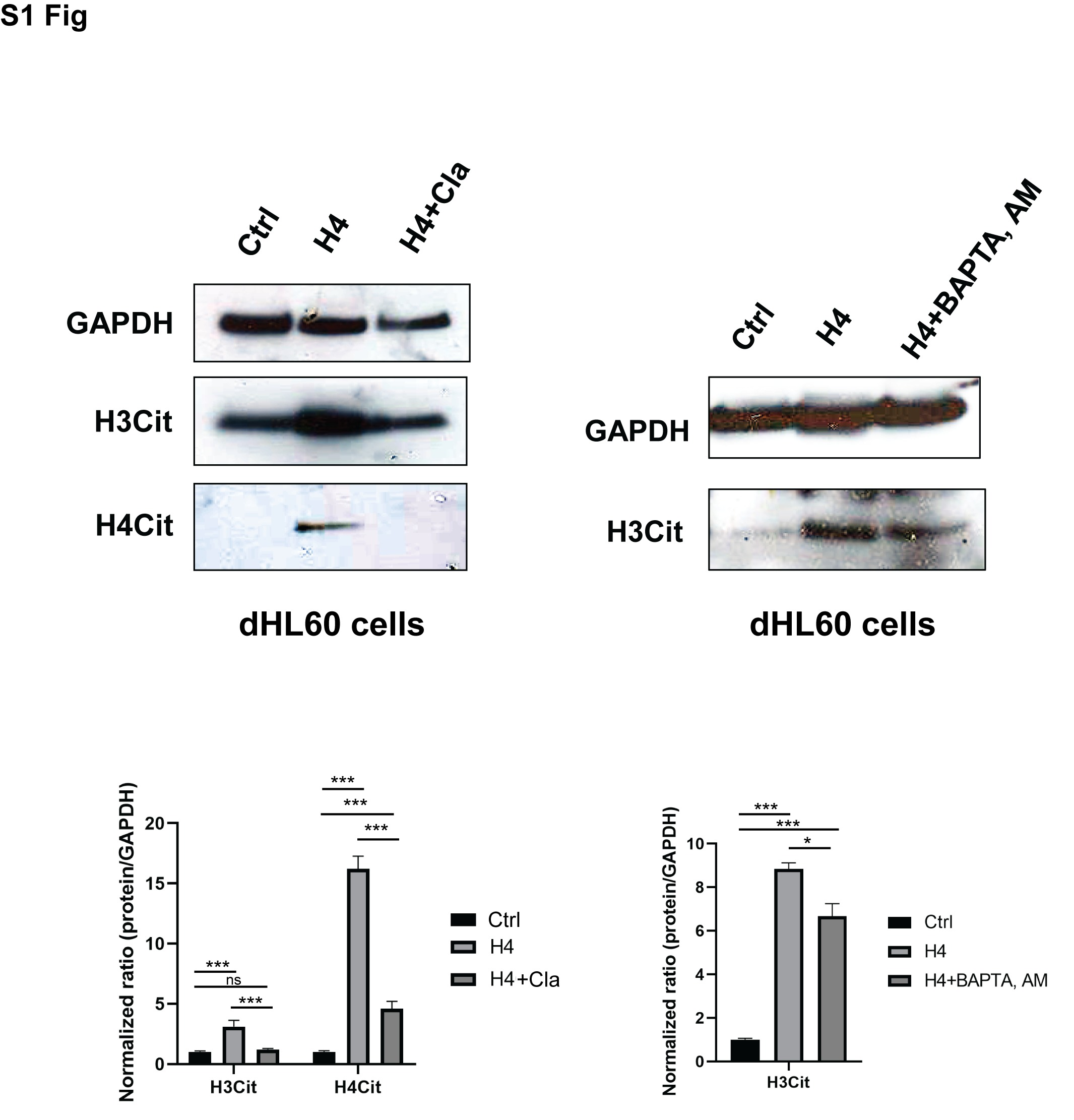

Supplement: S1 Fig — H3Cit and H4Cit protein levels were determined by western blot analysis in dHL60 cells after treatments with histone H4 or Cl-amidine + histone H4. The results are representative of three experiments. H3Cit protein level was determined by western blot analysis in dHL60 cells after treatments with histone H4 or BAPTA, AM + histone H4. The results are representative of three experiments. *, P < 0.05; ***, P < 0.001; ns, not significant. Ctrl, unstimulated control dHL60 cells. (TIF) [file pone.0251726.s001.tif]

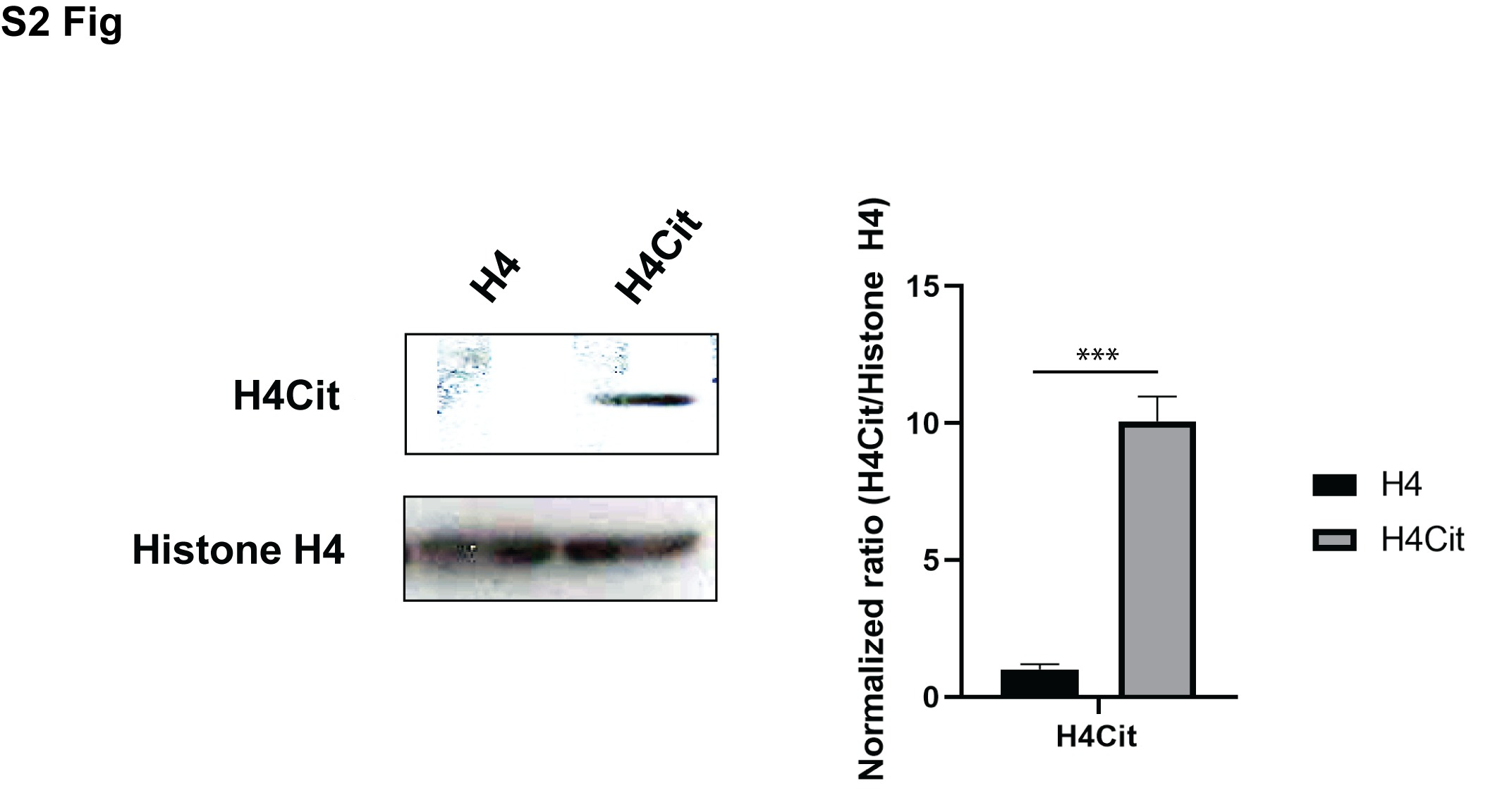

Supplement: S2 Fig — Citrullination of histone H4 with PAD4 was verified by western blot analysis. The results are representative of seven experiments. ***, P < 0.001. (TIF) [file pone.0251726.s002.tif]

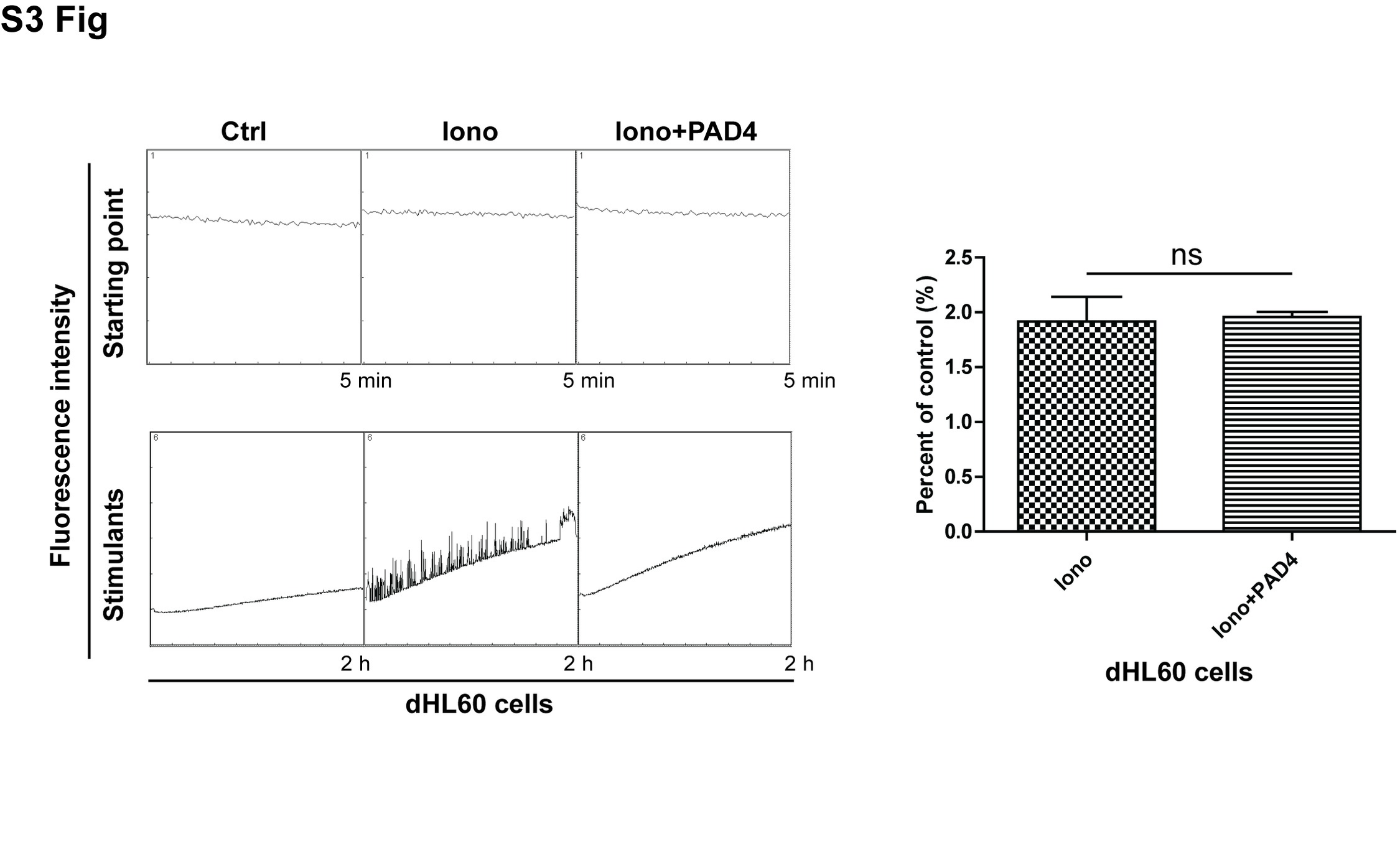

Supplement: S3 Fig — Fluo-4 kinetic fluorescent readings of dHL60 cells in resting condition (Starting point) and during 2 h of indicated treatments (Stimulants). The area under the curve from ionomycin or ionomycin + PAD4 treatments were normalized to control treatments for comparison. Mean ± SD is shown (n = 3 independent experiments); ns, not significant. (TIF) [file pone.0251726.s003.tif]

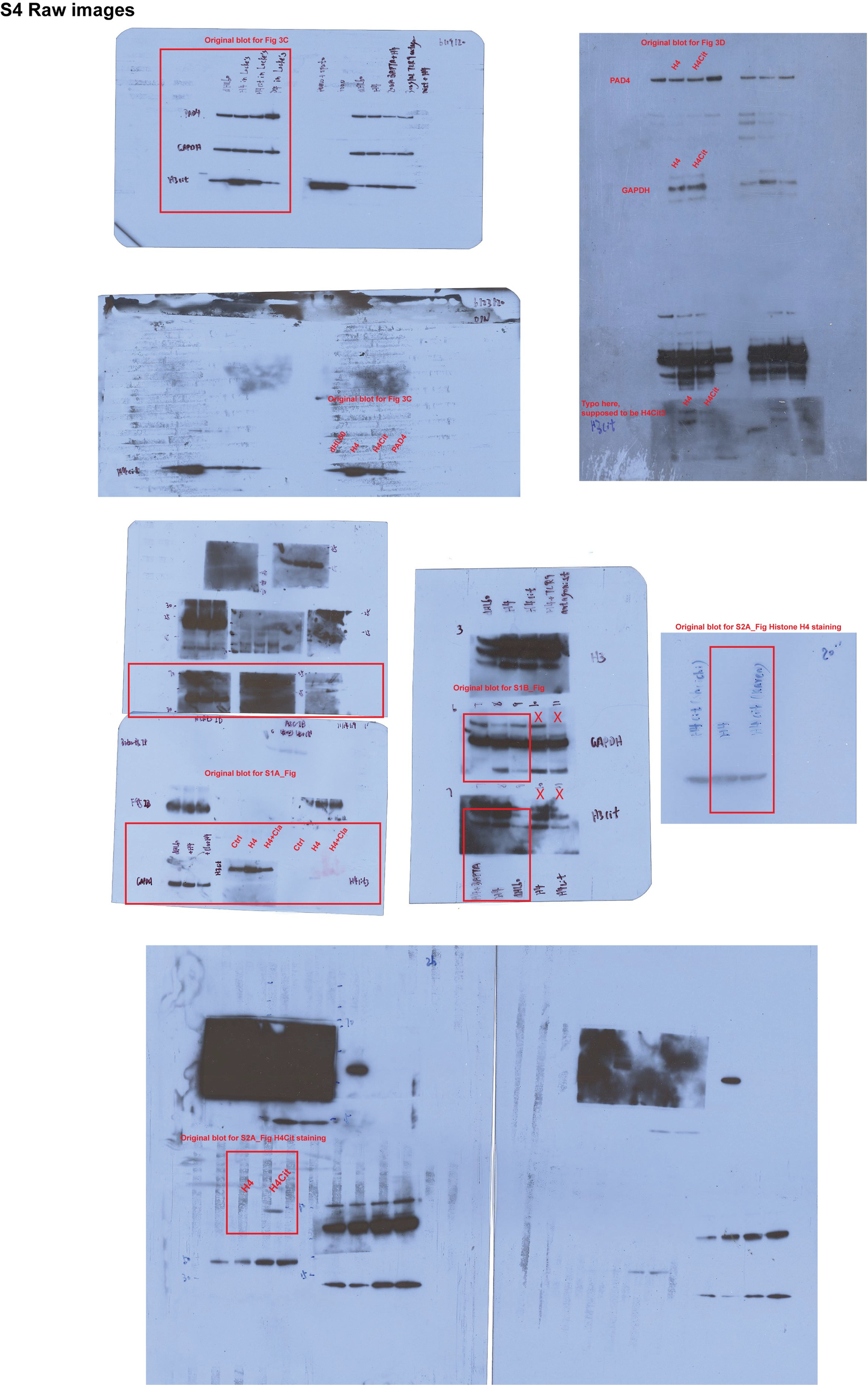

Supplement: S1 Raw images — (TIF) [file pone.0251726.s004.tif]
